# PI3K inhibitors in thrombosis and cardiovascular disease

**DOI:** 10.1186/s40169-020-0261-6

**Published:** 2020-01-31

**Authors:** Tom N. Durrant, Ingeborg Hers

**Affiliations:** 1 Department of Chemistry, University of Oxford, Oxford, OX1 3QZ UK; 2 School of Physiology, Pharmacology and Neuroscience, Biomedical Sciences Building, University Walk, Bristol, BS8 1TD UK

**Keywords:** Cardiovascular disease, Thrombosis, Platelets, Phosphoinositide 3-kinase, PI3K, Phosphoinositides, Cellular signalling

## Abstract

Phosphoinositide 3-kinases (PI3Ks) are lipid kinases that regulate important intracellular signalling and vesicle trafficking events via the generation of 3-phosphoinositides. Comprising eight core isoforms across three classes, the PI3K family displays broad expression and function throughout mammalian tissues, and the (patho)physiological roles of these enzymes in the cardiovascular system present the PI3Ks as potential therapeutic targets in settings such as thrombosis, atherosclerosis and heart failure. This review will discuss the PI3K enzymes and their roles in cardiovascular physiology and disease, with a particular focus on platelet function and thrombosis. The current progress and future potential of targeting the PI3K enzymes for therapeutic benefit in cardiovascular disease will be considered, while the challenges of developing drugs against these master cellular regulators will be discussed.

## Background

Cardiovascular diseases (CVDs) are a leading cause of mortality and morbidity worldwide [1]. Major causes of CVD-related deaths include ischaemic heart disease or stroke, for which arterial thrombosis is a key component [2]. Platelets play a critical role in arterial thrombosis, and antiplatelet therapy is therefore a frontline antithrombotic strategy. While platelets are essential for normal haemostasis, where localised thrombi stem bleeding and support repair at sites of vascular damage, excessive activation and accumulation of platelets in the vasculature may lead to blood vessel occlusion, which can result in myocardial infarction or stroke [3]. The role of platelets in disease can be more complex however, including contribution to inflammation, reperfusion injury, tumour progression and metastasis, and microbial infection, while metabolic conditions such as diabetes can lead to platelet hyperactivity [2, 4].

The cyclooxygenase (COX) inhibitor, aspirin (acetylsalicyclic acid), has served as a mainstay antiplatelet agent for decades, and acts via the inhibition of prostaglandin H_2_ generation, and thus prevention of the formation of the platelet activator, thromboxane A_2_ [5, 6]. Aspirin is often administered in combination with a drug targeting the major platelet G protein-coupled receptor for ADP, P_2_Y_12_, including thienopyridines such as clopidogrel and prasugrel, and the reversible cyclopentyl-triazolopyrimidine, ticagrelor [7, 8]. Other platelet receptors that represent current or potential targets for clinical intervention include the major integrin α_IIb_β_3_ to prevent fibrinogen binding required for thrombus inter-platelet bridges (e.g. abciximab, eptifibatide), protease-activated receptor (PAR) 1 or 4 for thrombin signalling (e.g. vorapaxar and atopaxar), and the collagen receptor Glycoprotein VI (GPVI) [3, 5, 9, 10]. While existing antiplatelet drugs offer considerable value for patients, the risk of unwanted bleeding associated with these therapies remains a major limitation, while individuals may also show a poor response to existing agents due to the specific nature of their metabolism; individuals with a reduced-function cytochrome P450 CYP2C19 allele are unable to efficiently metabolise clopidogrel to its active metabolite, for example [11]. A need for novel antithrombotic targets and associated drugs therefore remains. One such potential target is the phosphoinositide 3-kinase (PI3K) family, which comprises a range of lipid kinases that catalyse phosphorylation of the inositol ring of phosphatidylinositol (PtdIns) and its associated phosphoinositides to generate 3-phosphorylated lipid regulators of cell function. These enzymes are commonly activated downstream of the key clinically-targeted platelet receptors discussed above, to support platelet activation and thrombus formation, and may therefore represent promising candidates for the prevention of thrombosis. The PI3K enzymes are also implicated in other cardiovascular settings, including angiogenesis, hypertension and heart failure. This review will provide an overview of the PI3K enzymes, cover their roles in cardiovascular disease with a particular focus on thrombosis, and discuss the potential, progress and challenges of targeting this family of proteins for therapeutic means.

## The PI3K family

**Fig. 1:**
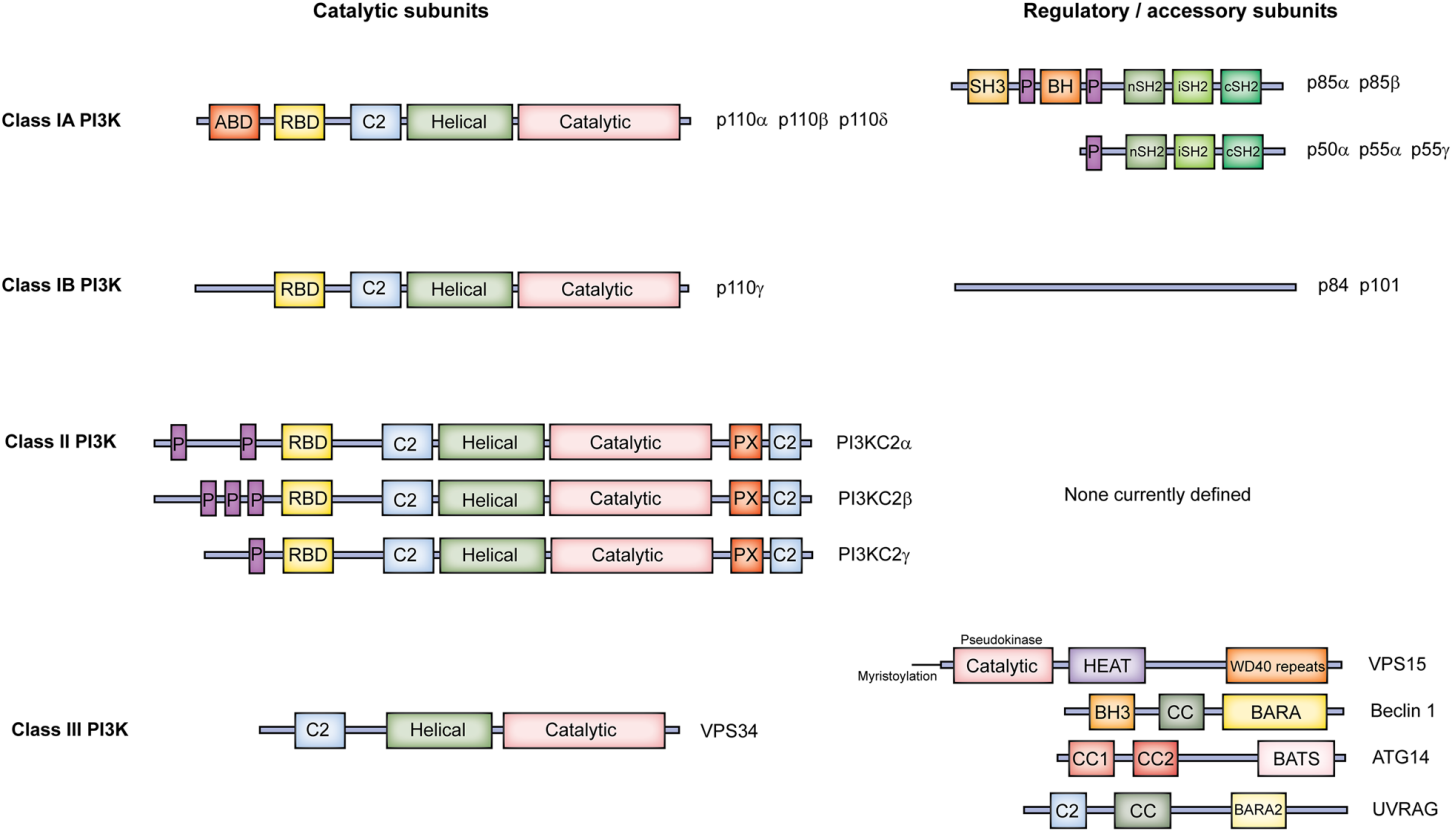
Domain organisation of the PI3Ks. Class I, II and III PI3K catalytic subunits share a core region consisting of a C2 domain, helical domain and kinase domain, but differ in other regions of the protein. While the domain structure of the Class IA regulatory subunits is well defined, the Class IB regulatory subunits have no clearly-defined domain structure, while no regulatory subunits have been reported for the Class II PI3Ks. The Class III PI3K, VPS34, associates with a range of accessory proteins to form at least two distinct complexes

PI3Ks catalyse phosphorylation of the 3-OH moiety of the inositol ring of PtdIns and its related phosphoinositides using the γ-phosphate of ATP, and regulate various important aspects of cellular function to support organismal physiology [12]. Mechanistically, this predominantly occurs via the ability of the 3-phosphoinositide products to regulate the localisation and activity of varied repertoires of effector proteins [13]. Mammals have eight core PI3K isoforms (catalytic-regulatory subunit pairing diversity increases this number, discussed later), divided into three classes based on structural, catalytic and regulatory properties (Fig. 1) [14]. The Class I PI3K family is the best characterised, and much is known about its members’ ubiquitous roles in receptor-initiated signal transduction in multiple cardiovascular tissues, and their significance in disease. Although their characterisation has lagged behind that of the Class I PI3Ks, the Class II and Class III PI3K families have received increased attention in recent years, and details of their organismal roles are now coming to light.

## Class I PI3Ks

**Fig. 2:**
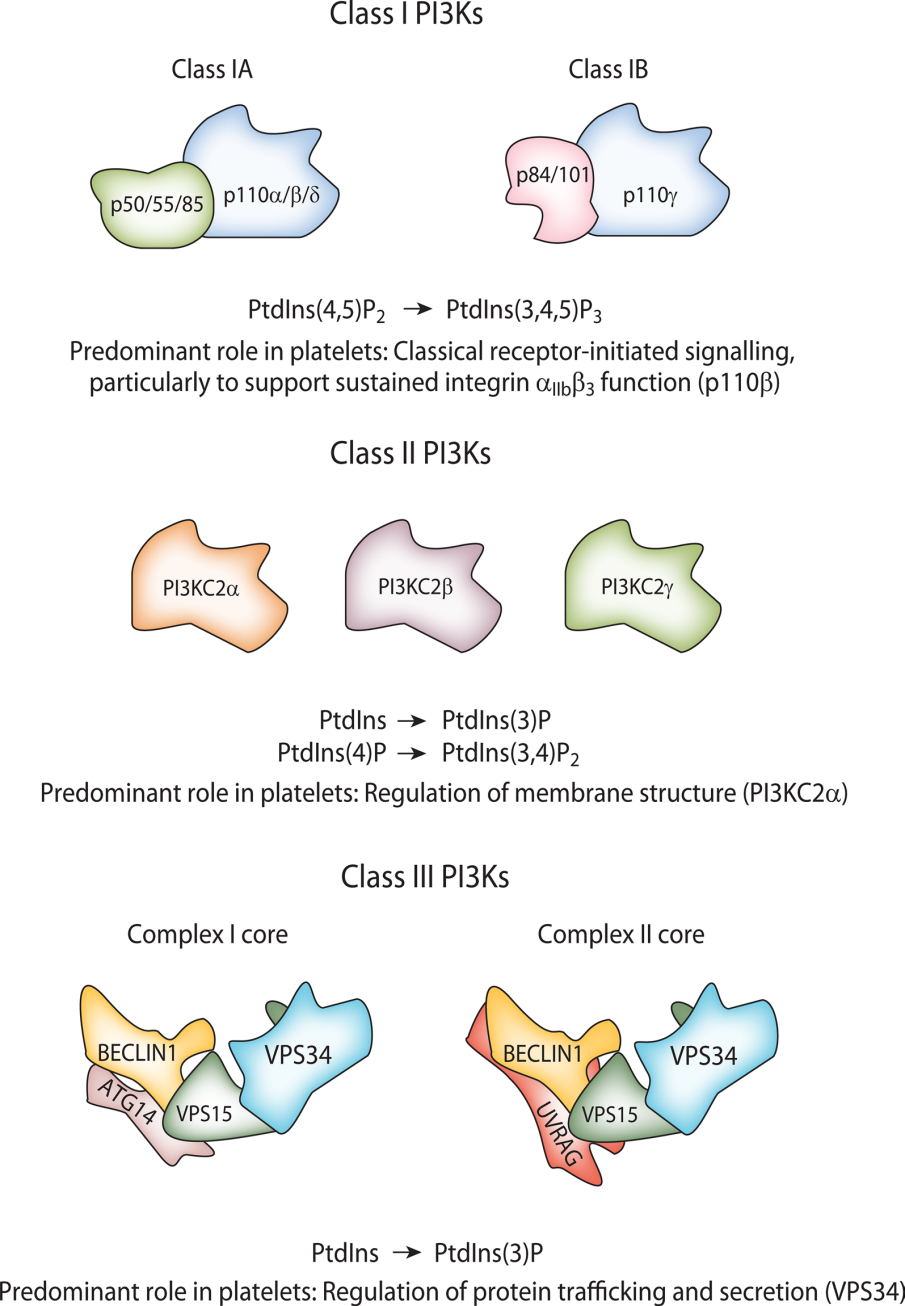
Overview of the PI3K complexes and their activities. Class I PI3Ks form heterodimers comprised of a catalytic subunit and a regulatory subunit, although free Class IA regulatory subunits also exist in cells. In vivo, Class I PI3Ks 3-phosphorylate PtdIns(4,5)P_2_ to generate PtdIns(3,4,5)P_3_. Class II PI3Ks appear to function as independent catalytic subunits, and are currently considered to 3-phosphorylate PtdIns and PtdIns(4)P in vivo to yield PtdIns(3)P and PtdIns(3,4)P_2_, respectively. However, Class II PI3K-deficient mouse models suggest PI3KC2α may only act to regulate a housekeeping pool of PtdIns(3)P in platelets, while PI3KC2β has no clear functional role, and PI3KC2γ is not expressed in this cell type. Class III PI3K, VPS34, associates with various proteins to form at least two known complexes, although it is likely that other associations and complexes exist. Class III PI3K phosphorylates PtdIns to generate PtdIns(3)P. Class I, II and III PI3Ks all play roles in platelet thrombus formation, and the predominant role of each is detailed

Class I PI3Ks are predominantly activated downstream of cell surface receptor stimulation to catalyse the phosphorylation of PtdIns(4,5)P_2_ to generate PtdIns(3,4,5)P_3_ [12]. These heterodimeric enzymes comprise a catalytic subunit, known as the p110, associated with a regulatory subunit [15]. The catalytic p110 subunit present in a Class I PI3K heterodimer defines the isoform nomenclature; i.e. PI3Kα, β, δ and γ isoforms contain p110α (PIK3CA gene), p110β (PIK3CB), p110δ (PIK3CD) or p110γ (PIK3CG), respectively. The Class I PI3K family is subdivided into Classes IA and IB, based on differing preferences of the catalytic subunits for regulatory partners (Fig. 1) [14]. The Class IA PI3K family comprises p110α, β and δ which can associate with the five regulatory subunits, p85α, p55α, p50α (PIK3R1 gene), p85β (PIK3R2) and p55γ (PIK3R3). In contrast, p110γ can associate with the p101 (PIK3R5) or p84 (PIK3R6) regulatory subunits to yield the Class IB PI3Ks (Fig. 2). Inter- and intra-subunit contacts allow regulation of the catalytic subunit to modulate the activity of these enzymes. For the Class IA PI3Ks, this includes important contacts between the N-terminal Src homology 2 (nSH2) and cSH2 domains of the p85 subunit and the regulatory arch within the kinase domain of the p110, although the cSH2 contact is lacking for p110α [14, 16, 17]. Although the structural composition of the Class IB PI3K regulatory subunits p101 and p84 remains more poorly defined than that of the Class IA regulatory subunits, these proteins appear to stabilise the C2-RBD (Ras-binding domain) linker and C2-helical linker of p110γ, respectively, while also stabilising p110γ’s helical domain [18, 19]. Activatory interactions between Class I PI3Ks and receptors or other proteins, discussed below, lead to a disruption of the inhibitory contacts within the PI3K heterodimers [14, 20]. Structural characterisation of these mechanisms not only advances understanding of fundamental Class I PI3K activation, but can also reveal the impact of mutations (e.g. oncogenic), and provides potential for novel approaches to drug design [20]. For example, building upon earlier structural studies [14], recent atomic level simulations have provided a detailed explanation of PI3Kα activation, revealing how loss of the interaction between the nSH2 of p85α and p110α initiates allosteric motions, with structural rearrangement reducing the distance between the ATP- and PtdIns(4,5)P_2_-binding sites in p110α to enable phosphoryl transfer [20]. Furthermore, observation of a deep cavity between the ATP- and substrate-binding sites in active PI3Kα suggests the potential for a novel approach to isoform-specific drug design [20], highlighting the value of structural studies of PI3K for drug discovery.

Studies to uncover the isoform-specific roles of the Class I PI3Ks have understandably focussed primarily on the catalytic p110 subunits using mouse gene targeting and small molecule catalytic inhibitors, while the identity and significance of the regulatory subunit in any given Class I PI3K heterodimer and context is often overlooked. Interestingly, recent work has demonstrated selectivity of Class IA PI3K composition in vivo, with p85α showing preferential association with p110δ [15], while Class IB PI3K family p110γ-p84 and p110γ-p101 heterodimers can fulfil distinct functional roles [21, 22]. In addition, p110-free p85 regulatory subunits exist within cells and can modulate PI3K activation, including a tumour suppressor role [15, 23, 24]. Specificity of heterodimer formation and function, and the presence of p110-free regulatory subunits, thus likely afford a currently underappreciated level of complexity and regulation to Class I PI3K function, which warrants further investigation and may offer new avenues for greater isoform and/or functional selectivity in drug design.

Mechanistically, selectivity of activation and function is known to be afforded to this enzyme family by the ability of the Class I PI3K isoforms to be differentially regulated by distinct sets of protein–protein interactions. The SH2 domains of the regulatory subunits of the Class IA PI3Ks allow interaction with phosphorylated tyrosines (often within the context of a ‘YXXM motif’) present on receptors or adaptor proteins [15]. In contrast, the Class IB PI3K regulatory subunits, p84 and p101, lack these SH2 domains but, like the catalytic subunit p110γ, can interact with Gβγ subunits to permit activation of PI3Kγ downstream of G protein-coupled receptors (GPCRs) [18, 25–27]. Class IA PI3Kβ can also be activated via a direct interaction of Gβγ subunits with p110β [28, 29], making it uniquely poised to respond to both (receptor) tyrosine kinase- and GPCR-directed signalling. Furthermore, PI3Kβ is unique among the Class I PI3Ks in its ability to respond to small GTPases, receiving activatory input via direct interaction with RHO-family RAC and CDC42 proteins [30] and RAB5 [31], while PI3Kα, PI3Kδ and PI3Kγ interact with RAS-family GTPases [32–34]. It is important to note however that, despite these isoform-specific properties, the interconnectivity of signalling events in cells, whereby different PI3Ks may be activated both directly and indirectly by overlapping and cross-talking sets of receptors and upstream regulators, can make it considerably challenging to dissect isoform-specific activities, as does potential isoform synergy and redundancy. Despite this, it would seem that PI3Kβ is well-suited to serve as a hub to receive and integrate signals from multiple inputs [35, 36], which may explain the dominance of this Class I PI3K isoform in platelets, where coincidence signalling is critical for cellular function [37].

Isoform-selective physiological roles for the Class I PI3Ks have emerged over the years, with PI3Kα holding a key role in embryonic development and angiogenesis [38–40], growth factor signalling [41], and insulin signalling and metabolism [42, 43], among other processes. The other broadly expressed Class I isoform, PI3Kβ, holds a key role in male fertility [44, 45], autophagy [46], immune complex-mediated neutrophil activation [35, 47], and platelet function [48], as discussed later. The functional significance of PI3Kδ and PI3Kγ is most apparent in cells of the myeloid and lymphoid lineages where these isoforms show highest expression, and includes the regulation of various aspects of inflammation and immunity, including B cell development, T cell differentiation and neutrophil migration [49–53]. While differential expression may offer broad explanations for some isoform-selective roles, in most scenarios several isoforms are present to some extent, and the specific details and context of the activatory stimulus often define the relative contribution of the different Class I PI3Ks in any given setting.

PtdIns(3,4,5)P_3_ generated by Class I PI3Ks serves to dictate cell function via the recruitment and regulation of various effector proteins. These effectors span a range of functional protein classes, including protein kinases and other enzymes, signalling adaptors, and regulators of small GTPases, thus allowing Class I PI3Ks to initiate and contribute to diverse signalling pathways inside cells [13]. PtdIns(3,4,5)P_3_ (and PtdIns(3,4)P_2_) effectors commonly possess a subtype of the pleckstrin homology (PH) domain, which interacts with the phosphorylated headgroup of this phosphoinositide via a conserved network of basic residues, although other protein domains can also interact with PtdIns(3,4,5)P_3_, including the DHR-1 domain of DOCK-family GEFs and the PX domain of sorting nexins [54–57]. While AKT (also known as Protein Kinase B) is the best characterised PtdIns(3,4,5)P_3_ effector and has received considerable attention in platelets [58], a range of others have key roles in this cell type, including RASA3 [59, 60], DAPP1 [13] and ELMO1 [61]. In highly dynamic cells like platelets, which utilise major cytoskeletal reorganisation events and protein trafficking during activation and thrombus formation, the ability of Class I PI3Ks to control small GTPases via a range of PtdIns(3,4,5)P_3_-regulated GAPs and GEFs is particularly interesting and warrants further investigation. Furthermore, the apparent ability of RAC and CDC42, which are key regulators of platelet function [62], to integrate both upstream (via interactions with the RBD of p110β) and downstream (via PtdIns(3,4,5)P_3_-regulated GEFs and GAPS) of PI3K suggests an intricate and tightly feedback-regulated signalling pathway in this cell type. PtdIns(3,4)P_2_ may support PtdIns(3,4,5)P_3_ signalling and can activate a subset of the same effectors, while it may also drive specific signalling, depending on the cell type and stimulatory context [63, 64]. The strength and duration of signalling is highly dependent on phosphatase activity, with 5′ phosphatases such as SHIP1 and SHIP2 dephosphorylating PtdIns(3,4,5)P_3_ to PtdIns(3,4)P_2_, while PTEN dephosphorylates PtdIns(3,4,5)P_3_ (and also PtdIns(3,4)P_2_) at the 3′ position of the inositol ring to yield PtdIns(4,5)P_2_ (and PtdIns(4)P) [65, 66]. In addition to the ability of Class I PI3Ks to regulate cellular behaviour via their catalytic activity, they can also contribute to cellular function via non-catalytic protein–protein interactions, commonly referred to as ‘scaffolding’ roles [67]. This explains divergence between animal studies with mice either lacking expression of Class I PI3K catalytic subunits, or expressing mutant kinase-dead forms, and is also an important consideration for therapeutic targeting using small molecules that may only inhibit the PI3Ks’ catalytic activity.

## Class I PI3Ks in platelet function and thrombosis

Human platelets express all Class I PI3K isoforms, with PI3Kβ expressed at the highest level followed by PI3Kγ > PI3Kα > PI3Kδ [68]. Of these, PI3Kβ has been revealed as the most important Class I isoform in platelet function and thrombosis, as demonstrated by the use of different mouse models and pharmacological approaches [69–74]. Deletion of p110β in megakaryocytes/platelets, or expression of a catalytically-inactive form, resulted in impaired platelet activation downstream of the collagen receptor GPVI and integrin α_IIb_β_3_, whereas the contribution of PI3Kβ to thrombin-mediated platelet activation was more modest [69, 71]. Interestingly, agonist-mediated production of PtdIns(3,4,5)P_3_ and downstream phosphorylation of AKT was largely impaired, demonstrating that PI3Kβ is the dominant isoform in raising intracellular PtdIns(3,4,5)P_3_ upon platelet activation [69, 71]. p110β-deficient conditional mice also showed impaired clot retraction and impaired in vivo thrombosis following FeCl(3) injury [71]. Although thrombus growth under physiological shear rate was not affected in p110β-deficient mice, at higher shear rates the formed thrombi showed enhanced embolization both ex vivo and in vivo [74]. Similar findings were obtained in ex vivo experiments using human blood treated with the PI3Kβ inhibitor AZD6482 [74]. This effect could be rescued by GSK3 inhibitors, suggesting that impaired activation of the AKT/GSK3 pathway may underlie thrombus instability [75]. Pharmacological approaches using selective p110β inhibitors such as TGX-221 and AZD6482 support the importance of PI3Kβ in platelet activation and thrombus formation, both in mouse and human studies [70, 72, 75–77].

Interestingly, the G_i_-coupled ADP receptor P_2_Y_12_ promotes PI3Kβ activation upon platelet stimulation, and supports platelet function by maintaining RAP1B activation, integrin α_IIb_β_3_ activation and aggregate stability, as well as promoting TxA_2_ formation through the ERK1/2 pathway [59, 73, 77]. In addition, P_2_Y_12_ signalling to PI3Kβ contributes to thrombin-mediated Ca^2+^ mobilisation and procoagulant activity [78]. Platelet primers such as thrombopoietin (TPO) can also synergistically increase PAR1-mediated RAP1B activation, integrin α_IIb_β_3_ activation and α-granule secretion, which was largely prevented by PI3Kβ inhibitors [79]. Furthermore, PI3Kβ contributes to the potentiation of platelet function by anti-phospholipid antibodies [80]. Indeed, multiple signalling inputs from different receptors, including P_2_Y_12_ and receptors that prime platelet function, can combine in their ability to activate PI3Kβ, thereby further promoting platelet function. The mechanisms by which PI3Kβ and other isoforms modulate platelet function have not been completely elucidated but are likely to involve signalling molecules regulated downstream of PtdIns(3,4,5)P_3_ and/or PtdIns(3,4)P_2_, including RASA3 [59, 60], DAPP1 [13], ELMO1 [61] and AKT/GSK3 [74, 81, 82].

The PI3Kγ isoform has also been shown to support platelet activation downstream of the ADP receptor P_2_Y_12_. The aggregation response to ADP, but not collagen and thrombin, was significantly reduced in platelets deficient in PI3Kγ [83]. Furthermore, these mice were protected against ADP-dependent thromboembolic vascular occlusion [83]. Interestingly, both PI3Kγ and PI3Kβ are required for maintaining integrin α_IIb_β_3_ activation and platelet aggregate stability, as determined in aggregation and ex vivo and in vivo thrombosis models [73, 84], with the contribution of PI3Kγ potentially mediated through a non-catalytic signalling mechanism [84]. Furthermore, dual activation of PI3Kγ and PI3Kβ underlies the ability of TPO to enhance platelet function through the ERK1/2/TxA_2_ pathway [79].

PI3Kα has a more subtle role in platelet function, with initial studies using the PI3Kα selective inhibitor, PIK75, reporting a role for PI3Kα in IGF1-mediated enhancement of platelet function [85, 86]. Furthermore, inhibitor studies showed that PI3Kα may also contribute to GPVI-mediated platelet function [86, 87]. Two groups, including our own, subsequently generated a mouse model where p110α has been selectively deleted in the megakaryocytic lineage [88]. Combining genetic and pharmacological approaches, including careful titration of PI3Kα and β inhibitors, we revealed that both PI3Kα and PI3Kβ contribute to IGF1-mediated AKT phosphorylation, but that PI3Kα is the isoform responsible for supporting platelet function [88]. PAR4-, thrombin-, CRP- and fucoidan-mediated integrin α_IIb_β_3_ activation and α-granule release, as well as thrombus formation on a collagen-coated surface under flow, were not affected [88]. In contrast, p110α deletion, but not PI3Kα inhibition, resulted in a synergistic enhancement of TPO-mediated priming of platelet function by increasing ERK1/2 phosphorylation and TxA_2_ formation, suggesting a novel kinase-independent negative regulatory role for PI3Kα in platelet function. Laurent et al. [89] also found more discrete roles for PI3Kα in platelet function, with PI3Kα deficiency and inhibitors reducing ADP secretion at low levels of GPVI activation and impairing platelet adhesion to vWF under shear. PI3Kα, together with PI3Kβ, also contributes to the platelet priming effect of anti-phospholipid antibodies [80]. More importantly, PI3Kα deletion and inhibition resulted in decreased arterial thrombosis without affecting bleeding time, suggesting it has potential as an anti-thrombotic target [89].

The PI3Kδ isoform is expressed at low levels in both mouse and human platelets [68, 90] and plays only a minor role in platelet function [91]. p110δ-deficient platelets, or mouse platelets expressing a catalytically-inactive form of p110δ, showed minor aggregation defects, reduced spreading on fibrinogen and vWF, but normal thrombus formation on collagen under flow [91].

## Class II PI3Ks

Class II PI3Ks are currently considered to catalyse phosphorylation of the 3′ position of the inositol ring of PtdIns or PtdIns(4)P in vivo to generate PtdIns(3)P or PtdIns(3,4)P_2_, respectively [92, 93]. Achieving unambiguous confidence in the relative contributions of PI3Ks to the turnover of specific phosphoinositides in vivo is however highly challenging, due to the complexity of the interconnected phosphoinositide network, and due to technical difficulties in measuring specific lipids. It also appears that the relative production of PtdIns(3)P or PtdIns(3,4)P_2_ by Class II PI3Ks may depend on the local abundance of their respective substrate at the site of action, such as plasma or endosomal membranes, and on the cell type studied [93, 94].

The Class II PI3K family comprises three isoforms, PI3KC2α (PIK3C2A gene), β (PIK3C2B) and γ (PIK3C2G), which appear to function as catalytic monomers, without regulatory partners (Fig. 1) [93]. Similar to the Class I PI3K p110s, these enzymes possess RBD, C2, helical and catalytic domains, but also possess a poorly-structured N-terminal region, in addition to a C-terminal region containing C2 and PX domains [14, 95]. Understanding of Class II PI3K regulation is in its infancy, but for PI3KC2α, the N-terminal region has been shown to support plasma membrane recruitment of the enzyme via interactions with clathrin [96, 97], while the C2 and PX domains interact with phosphoinositides such as PtdIns(4,5)P_2_ to release an autoinhibitory mechanism to enable catalytic activity [98–100]. It is likely that multiple further interactions with proteins and lipids can regulate the function of the Class II PI3Ks and, as for the Class I PI3Ks, specificity within such interactions is likely to guide differential usage of the three Class II isoforms [93]. While PI3KC2γ expression appears to be largely restricted to tissues such as liver, breast and prostate, PI3KC2α and PI3KC2β demonstrate more widespread expression [101–103]. In a manner analogous to Class I PI3K-generated PtdIns(3,4,5)P_3_, the Class II PI3K products PtdIns(3)P and PtdIns(3,4)P_2_ can regulate cell function via a range of effectors containing 3-phosphoinositide-binding elements such as FYVE, PX and PH domains [104].

Class II PI3Ks predominantly serve to modulate cell function via the regulation of membrane trafficking and dynamics [93], and the lethality of PI3KC2α loss or inactivation in mice demonstrates the critical role of this Class II isoform in embryonic development [105–107]. Indeed, PI3KC2α has been shown to regulate angiogenesis, insulin signalling and glucose transport, sonic hedgehog signalling, primary cilium assembly and clathrin-mediated endocytosis [93, 94, 105, 107–109]. The impact of homozygous loss of PI3KC2α appears to be less severe in humans, potentially reflecting differential usage of Class II PI3Ks between humans and mice, or a differing ability to compensate [110]. Such species difference is an important factor in the consideration of PI3K inhibitor development for human disease. Loss of PI3KC2β or PI3KC2γ expression or activity in mice yields viable animals, albeit with distinct metabolic phenotypes of increased or decreased insulin sensitivity, respectively [101, 111, 112]. Furthermore, heterozygous loss of PI3KC2α activity induces mild, age-dependent obesity, insulin resistance and glucose intolerance, in addition to leptin resistance in male mice, although females display no metabolic phenotype [106]. PI3KC2α’s role in endocytosis includes the synthesis of a local pool of PtdIns(3,4)P_2_ at late-stage endocytic intermediates to recruit SNX9 to support dynamin-mediated membrane scission for vesicle release from the invaginated membrane, while this class II PI3K also supports the removal of recycling cargo from endosomes via the production of PtdIns(3)P and the activation of RAB11 [97, 107, 113]. Class II PI3KC2β has an important role in mTORC1 regulation on lysosomes and late endosomes [114], in regulation of the potassium channel KCa3.1 in CD4 T-cells and mast cells [115, 116], in mitosis progression [117] and in cell migration [118–120]. Class II PI3K’s can hold non-catalytic scaffolding roles, including a role for PI3KC2α in mitotic spindle stabilization during metaphase [121], with such roles potentially being resistant to inhibition with small molecules targeting the catalytic activity of these enzymes.

## Class II PI3Ks in platelet function and thrombosis

Human and mouse platelets express PI3KC2α and β, but not PI3KC2γ [122]. Due to the lethality of PI3KC2α loss in mice, mouse studies have utilised heterozygous knock-in of a kinase-dead mutation (D1268A) at the endogenous locus [123], or inducible shRNA gene targeting [122, 124, 125]. PI3KC2α-deficient mouse platelets show defective thrombus formation, forming accelerated but highly unstable thrombi under haemodynamic shear [122, 123]. Interestingly, although there was a decrease in a ‘housekeeping’ pool of PtdIns(3)P, the platelet phenotype observed with loss of PI3KC2α was not associated with defects in agonist-induced changes in PtdIns3P or PtdIns(3,4)P_2_. Instead, PI3KC2α-deficient platelets displayed various structural and biophysical changes in their membranes, including an enlarged open canalicular system (OCS), enhanced membrane tethers, an enrichment of barbell proplatelets, a reduction in certain membrane skeleton proteins, decreased filopodia, and defects in α-granules [122, 123]. Megakaryocytes (MKs), large cells residing in the bone marrow involved in platelet production, also showed an abnormal demarcation membrane system, confirming the membrane defects not to be specific to platelets, and defining an important role for PI3KC2α in membrane structure and dynamics [92, 122, 123].

Although an early study suggested a role of PI3KC2β in PtdIns(3,4)P_2_ generation following integrin α_IIb_β_3_ activation [126], platelets from PI3KC2β-deficient mice showed unaltered basal or agonist-stimulated levels of PtdIns(3)P, PtdIns(3,4)P_2_ or PtdIns(3,4,5)P_3_ compared to wild type littermates [122]. Furthermore, PI3KC2β-deficient platelets had normal platelet functional, haemostatic and thrombotic responses [122]. Loss of both PI3KC2α and PI3KC2β had no impact on agonist-stimulated 3-phosphoinositide levels, and confirmed that PI3KC2α’s role in the regulation of platelet open canalicular structure and thrombus stability is non-redundant, although VPS34 expression was increased in this context [125]. Subsequent work using ion beam-scattering electron microscopy and mass spectrometry confirmed that the defect in platelet membrane structure observed for PI3KC2α-deficient platelets is not associated with major changes in membrane lipid composition, but is due to increased OCS dilation, volume, and plasma membrane openings, with a potential impact on membrane tethering during thrombus formation [124]. It is important to note that a lack of selective inhibitors for the Class II PI3Ks has hampered further interrogation of their functional roles in many contexts, including whether the functional significance observed in mice will translate fully to humans. However, Class II PI3K inhibitors are beginning to emerge, and may be of value in thrombosis, cancer and other settings, as discussed below [117, 127–129].

## Class III PI3K

The sole Class III PI3K, VPS34 (PIK3C3 gene), is the primordial PI3K conserved across species from yeast to humans, and serves to catalyse the generation of PtdIns(3)P from PtdIns [130–132]. VPS34 is widely expressed across mammalian tissues, and forms two protein complexes, Complex I and Complex II (Fig. 1) [93, 133]. In addition to VPS34, both complexes contain VPS15 (PIK3R4) and Beclin 1, although Complex I also contains ATG14, whereas Complex II contains UVRAG (Fig. 2) [134]. Complex I may also incorporate the regulatory proteins NRBF2 or AMBRA, while Complex II can contain Rubicon, although it appears likely that several VPS34-containing complexes of varying composition may exist in cells [93]. The helical and kinase domains of VPS34 are flexible and regulate its catalytic activity by adopting closed or open conformations, as the C-terminal helix blocks the ATP-binding site until the association of the helix with the membrane removes this inhibition [135]. The helical and kinase domains of VPS34 are positioned on one side of a V-shaped assembly that both Complex I and II appear to form, and interact with VPS15 [14, 93]. Beclin 1, and ATG14 or UVRAG, are positioned on the other arm of the V assembly, which also mediates membrane association (Fig. 2) [14, 93]. ATG14 preferentially associates with highly curved membranes to facilitate the association of Complex I with the growing autophagic isolation membrane, while Complex II associates with relatively flat endosomal membranes potentially due to flexibility between the two arms of its V shape allowing an extended conformation [14, 136, 137]. Class III PI3K is highly regulated by post-translational modifications, including acetylation, ubiquitination, SUMOylation, and phosphorylation by enzymes such as AMPK and mTORC1, which can serve to modulate its catalytic activity and protein–protein interactions [14, 134].

Complex I’s activation and recruitment to the isolation membrane leads to PtdIns(3)P generation to support autophagosome formation and elongation, while Complex II supports endosome maturation and fusion of autophagosomes with late endosomes/lysosomes [93, 134]. As discussed earlier, Class II PI3Ks can also generate PtdIns(3)P, as can lipid phosphatases, and so Class III PI3K is not the sole source of this phosphoinositide in cells, and indeed its contribution appears to vary between cell types [93].

Various strategies have been utilised to assess Class III’s physiological role in differing tissues, including a kinase-dead mouse knock-in approach, knock-out approaches and, more recently, the use of inhibitors. Global homozygous loss of VPS34 catalytic activity or expression leads to embryonic lethality in mice between E6.5 and E8.5 [133, 138]. A corresponding impact on the expression of VPS34 interactors such as VPS15 in these models makes protein-specific phenotype interpretation more challenging, but the comparable embryonic lethality of knock-out and kinase-dead knock-in mice supports a role for this enzyme in early embryonic development, with defects in cell proliferation and mTOR signalling [133, 138]. Tissue-specific targeting has allowed further insight into the physiological roles of VPS34, with loss of VPS34 leading to cardiomyopathy and cardiomegaly in the heart [139, 140], hepatomegaly and hepatic steatosis in the liver [139], rod cell degeneration in the retina [141], neurodegeneration in the nervous system [142–144], and defective T cell survival and homeostasis [145, 146]. Many of these phenotypes were associated with defects in cellular autophagy or endocytic trafficking.

## Class III PI3K in platelet function and thrombosis

Both mouse and human platelets express the Class III PI3K protein, VPS34. Two detailed studies assessing mice with targeting of VPS34 in their megakaryocytes and platelets have revealed the functional importance of this enzyme in this cell lineage [147, 148]. The core phenotypes of the distinct VPS34-deficient mouse lines were comparable in that loss of Class III PI3K had no effect on haemostasis, but resulted in defective in vivo and in vitro thrombosis. Both studies reported smaller thrombi under arterial flow conditions, suggesting defects in thrombus growth and stability. This implies that VPS34 might hold value as an antithrombotic target, as discussed later, which has been supported by the use of the VPS34 inhibitors 3-MA, VPS34-IN1 and SAR405 in human platelets [147, 148].

However, the two VPS34-deficient mouse lines did differ in the characterisation of various specific MK and platelet features, potentially due to differences in experimental conditions or gene-targeting approaches and their relative penetration. While Liu et al. [147] reported a normal platelet count and morphology, Valet et al. [148] observed microthrombocytopenia, with a reduction in both platelet count and volume, and multiple phenotypic alterations in VPS34 deficient-megakaryocytes. The latter includes fewer but larger α-granules in MKs in native bone marrow, and increased release of platelets outside of the sinusoids directly into the bone marrow compartment. This ectopic platelet release is likely to underlie the thrombocytopenia in these mice [148]. In addition, VPS34-deficient MKs showed reduced transferrin and fibrinogen endocytosis, and decreased number and increased size of fibrinogen-containing and clathrin-coated vesicles, suggesting a defect in clathrin-mediated endocytosis [148]. RAB11 labelling also suggested impaired endosomal recycling, with VPS34-deficient MKs therefore demonstrating a general trafficking defect [148]. VPS34-deficient MKs showed a 30–40% reduction in PtdIns(3)P which, although confirming Class III PI3K to be a significant source of this phosphoinositide in this cell type, confirms the importance of other enzymatic routes of PtdIns(3)P synthesis in MKs [148].

Valet et al. [148] also revealed VPS34-deficient platelets to show multiple specific defects, many of which correspond to their observations in VSP34-deficient MKs. Although VPS34-deficient platelets showed reduced numbers of α and dense granules, their granule release was faster and exacerbated in response to acute platelet stimulation both in platelet suspensions and in vitro flow studies. VPS34-platelets were however less efficient in recruiting wild type platelets to allow further thrombus growth [148]. As for MKs, VPS34 is also likely to be involved in clathrin-mediated endocytosis in platelets, as platelet Mpl endocytosis and fibrinogen internalisation were both defective [148]. Indeed, the Pf4-Cre-*Pik3c3* mice showed elevated serum thrombopoietin (TPO), correlating with the reduced platelet count, and defective platelet Mpl endocytosis [148]. The contribution of VPS34 to total PtdIns(3)P levels in platelets was modest with a 10% decrease in PtdIns(3)P in VPS34 deficient platelets under resting conditions. Although the agonist induced pool of PtdIns(3)P was more markedly affected, the reduction was still partial, supporting the role of other enzymes in PtdIns(3)P generation in platelets [148]. Platelet shape change, filopodia formation, integrin activation, aggregation, ROS production and Thromboxane A_2_ production responses to a range of agonists were normal in VPS34-deficient murine platelets and in human platelets treated with a VPS34 inhibitor [148].

While the overall phenotype of VPS34-deficient mice was similar in the study by Liu et al. [147] a range of differences in platelet characteristics and responses were observed. In contrast to Valet et al. the number of platelet α and dense granules was normal in VPS34-deficient platelets. However, α and dense granule secretion, integrin α_IIb_β_3_ activation and platelet aggregation were defective in response to collagen and thrombin, in particular at lower agonist concentrations, while downstream phosphorylation of SYK and PLCγ2 (but not other pathways) was affected [147]. Furthermore, clot retraction of VPS34-deficient platelets was delayed, despite platelet spreading on fibrinogen and integrin β_3_ and SRC phosphorylation being normal, suggesting a defect in later, but not early, integrin outside-in signalling [147]. Interestingly, PtdIns(3)P levels were comparable between wild type and VPS34-deficient platelets, although VPS34-deficient platelets had a significantly lower response to thrombin or convulxin stimulation [147]. The partial effect of VPS34 deficiency on PtdIns(3)P levels is in agreement with Valet et al. [148] and studies investigating platelet PI3KC2α [123], and confirms the involvement of multiple enzymes in platelet PtdIns(3)P synthesis.

Interestingly, Liu et al.’s [147] findings also revealed that VSP34 supports NADH/NADPH oxidase (NOX) activity and subsequent generation of reactive oxygen species (ROS) to impact on platelet activation. VPS34-deficient platelets had reduced agonist-induced translocation of the NOX subunits p40phox and p47phox to the plasma membrane, p40phox phosphorylation and ROS generation [147]. VPS34 deficiency furthermore impaired mTORC1 and 2 activation, as judged by substrate phosphorylation, although this did not appear to influence platelet function. Similarly, although loss of VPS34 affected basal autophagic flux in resting platelets, with increased LC3-II in VPS34-deficient platelets, VPS34 did not hold an important role in autophagic flux associated with platelet activation, and the effects of autophagy inhibition did not match the phenotype of VPS34 loss [147]. Therefore, while loss of VPS34 function appears to drive defects in many tissue types due to an impact on autophagy, the phenotype of VPS34-deficient platelets does not appear to be solely driven by loss of this cellular process, despite potential importance for autophagy in platelets and the suggestion in other studies that its disruption has consequences for haemostasis and thrombosis [149, 150].

## PI3Ks as clinical targets for thrombosis

**Fig. 3:**
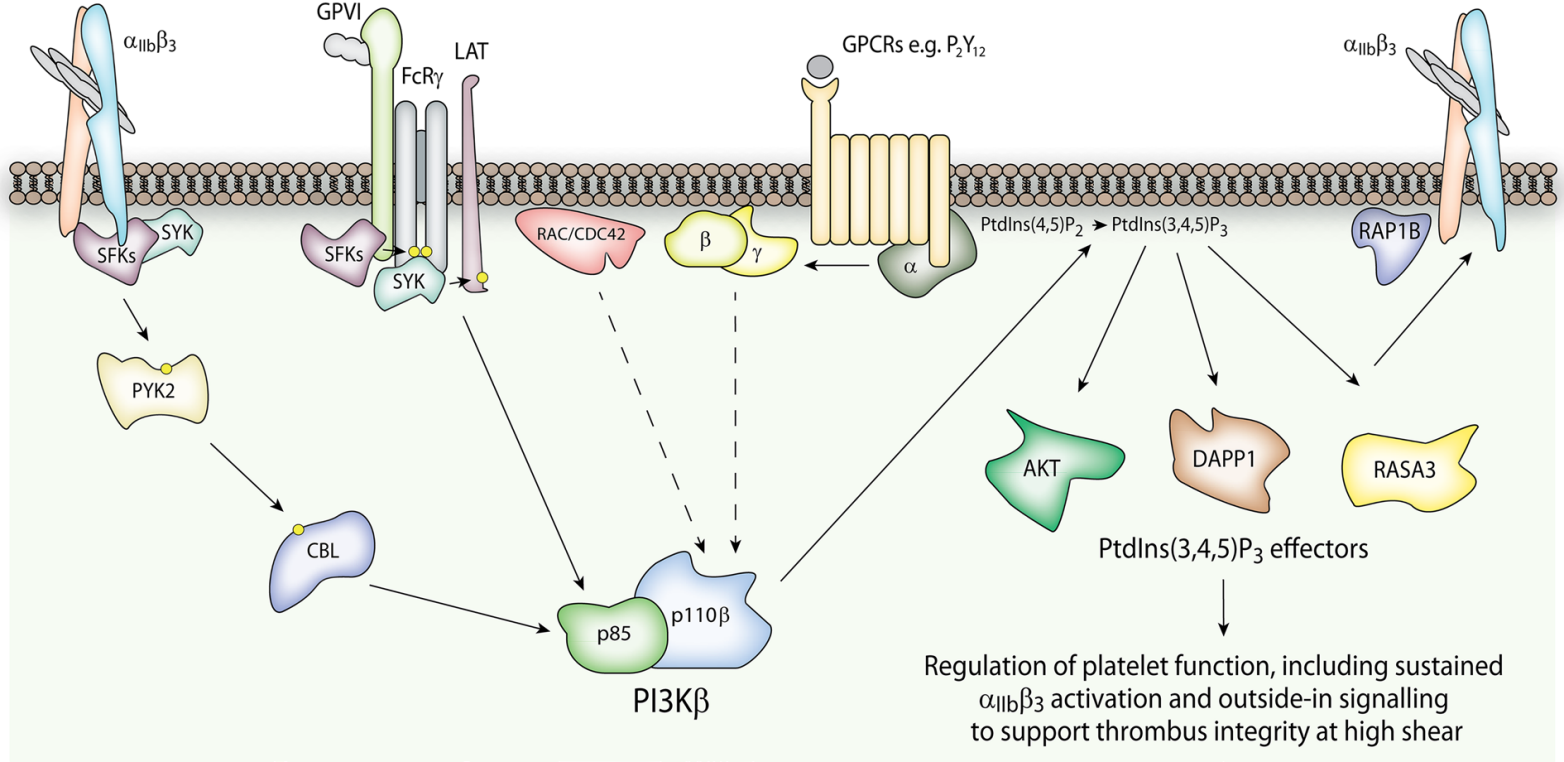
The role of PI3Kβ in platelets. Class I PI3Kβ is considered a potential antithrombotic target due to its functional role downstream of various platelet receptors, and has received the most intense clinical interest of the PI3Ks so far in this setting. In particular, it plays a key role in sustaining integrin α_IIb_β_3_ signalling to support stable thrombus formation under high shear, acting via the regulation of various cellular effectors which are responsive to its catalytic product, PtdIns(3,4,5)P_3_. PI3Kβ can receive activatory input via multiple interactions, including direct interaction of p85 with CBL downstream of α_IIb_β_3_, association of the SH2 domains of p85 with phosphorylated tyrosines on receptors and adaptors downstream of Glycoprotein VI (GPVI), and interactions of p110β with RAC/CDC42 and the Gβγ subunits of activated heterotrimeric G proteins downstream of GPCRs such as P_2_Y_12_

PI3K inhibitors have been in development for many years, driven by the therapeutic potential of targeting these enzymes in cancer, inflammatory and immune conditions. First generation compounds such as Wortmannin and LY294002 were limited by pan-PI3K inhibition and off-target action against other cellular kinases but have proven to be valuable tools for characterising PI3K signalling, while subsequent PI3K inhibitors with isoform-selectivity and/or improved pharmacology have received more serious consideration in the clinic in recent years [151–153]. To date, the focus of efforts to clinically target PI3Ks in thrombosis has been Class I PI3Kβ. This is because the Class I PI3Ks have received considerably more attention than Class II or III in this area so far, and because, as discussed above, PI3Kβ is the predominant functional Class I PI3K in platelets. Indeed, platelet PI3Kβ was the target of one of the earliest isoform-selective PI3K inhibitors, TGX-221 [70, 154]. The highly homologous nature of the ATP binding pocket of the Class I PI3Ks makes achieving isoform-selective inhibitors a major challenge, but the observation of two clusters of non-conserved residues at its periphery, and a hard-won understanding of the intricate details of the conformational flexibility and interactions of the binding pocket, have aided the development of inhibitors with impressive selectivity [155]. The use of TGX-221 defined a role for platelet PI3Kβ in initiating and sustaining α_IIb_β_3_ adhesive contacts, most notably under conditions of shear stress, thus proposing PI3Kβ as a new antithrombotic target (Fig. 3) [70]. This was subsequently supported and extended by a wide body of work using TGX-221 and gene-targeted mice, defining roles for PI3Kβ downstream of various platelet receptors to support thrombus formation in vivo, and confirming that PI3Kβ inhibition provides protection from arterial thrombosis, with limited effect on normal haemostasis [48, 69, 71, 75].

Based on this, development of further small molecules targeting PI3Kβ led to the derivation of AZD6482, which is an active enantiomer of a racemic mixture first known as KN-309, an improved structural analogue of TGX-221 [156]. AZD6482 has nanomolar potency against PI3Kβ and is highly selective for this enzyme against a panel of protein kinases, with lowest selectivity against the other Class I PI3Ks, and the related DNA-PK [72]. AZD6482 inhibits agonist- and shear-induced human platelet aggregation, and displayed a concentration-dependent anti-thrombotic effect in vivo in a modified Folt’s model in dogs, with no detectable increase in bleeding time or blood loss even at concentrations considerably higher than required for antithrombotic effect [72]. A 3 h infusion in healthy human male Caucasian subjects confirmed a maximal inhibition of platelet aggregation at 1uM in ex vivo assays, with limited effect on cutaneous bleeding time [72]. AZD6482 demonstrated a mean effective elimination half-life of ~ 20 min for the highest dose groups, and a rapid normalization of platelet function post end of infusion [72]. This was attributed to high clearance and a relatively small distribution volume, with the study authors proposing that AZD6482 may provide value as a parenteral antiplatelet agent in acute stroke where rapid onset of action and low bleeding risk is desirable, and in cardiothoracic surgery. Since extracorporeal circulation can lead to platelet dysfunction, and TGX-221 has been shown to be of value in this setting [157, 158], PI3Kβ inhibitors may be of use in cardiopulmonary bypass surgery, and avoid the bleeding risk associated with integrin α_IIb_β_3_ inhibitors [156]. While AZD6482, like other PI3K inhibitors, has shown some impact on glucose homeostasis, the study authors concluded that this would not be of clinical importance during short-term infusion as an antiplatelet agent [72], although on the basis that inhibition of PI3Kα may underlie any major effect on insulin signalling, PI3Kβ inhibitors with an improved selectivity ratio against PI3Kα are being actively sought [76, 159]. However, even with highly selective agents, it remains unclear whether long-term PI3Kβ inhibition beyond acute usage would be a viable therapeutic approach given PI3Kβ’s roles in multiple aspects of physiology, and considering mice with loss of PI3Kβ activity develop mild insulin resistance with age [160]. Despite this, PI3Kβ inhibitors are under development in cancer and may be of value in contexts of PTEN loss and genomic aberrations of the PI3Kβ locus [161, 162] and studies to date suggest these agents may have an acceptable safety profile, although this remains a major challenge for the development of Class I PI3K inhibitors in cancer (discussed later).

Since anti-platelet therapy is often administered as a combination of drugs, with Aspirin prescribed alongside a P_2_Y_12_ antagonist in settings where single therapy is insufficient, Nylander et al. [163] extended on their initial validation of AZD6482 with a study administering this drug as part of combination therapy. AZD6482 was assessed alongside the P_2_Y_12_ antagonists ticagrelor or clopidogrel, or alongside aspirin, in studies using dogs and healthy humans. Assessment of ex vivo antiplatelet effect using a conscious dog model confirmed the attractive profile of PI3Kβ inhibition in demonstrating anti-platelet efficacy with limited effect on bleeding time, while with healthy male Caucasian human subjects a combination of PI3Kβ inhibition plus COX inhibition with aspirin provided less bleeding potential, but more potential for greater overall anti-platelet effects, than P_2_Y_12_ inhibition plus aspirin [163]. This study therefore suggested that PI3Kβ inhibition could be of value not only as a monotherapy, but in combination with Aspirin, suggesting further clinical investigation of this enzyme as an anti-thrombotic target should take place.

Despite this promise for PI3Kβ as an anti-thrombotic target, its clinical potential may be hampered by the observation that its inhibition may lead to increased risk of embolism of thrombotic material [75, 164]. Laurent et al. [75] demonstrated a key role for PI3Kβ, via a AKT-GSK3 axis, in thrombus stability and recruitment of new platelets to the growing thrombus at high shear rate, observed using mice with selective loss of PI3Kβ in the megakaryocyte lineage and human platelets treated with AZD6482. The thrombus instability with loss of PI3Kβ activity at high shear rate was associated with the formation of platelet emboli from large thrombi, suggesting the potential for secondary ischaemic events, with the growing thrombus itself likely contributing to the elevation of local shear in the blood vessel [75, 164]. Further studies are needed to determine whether this property would rule out PI3Kβ inhibitors for clinical use, particularly since P_2_Y_12_ inhibition may cause a similar effect which has not limited its use clinically [156], and tailoring of PI3Kβ inhibitor dosage may mean a suitable level of inhibition can be found [164].

As an understanding of the role of Class II and Class III PI3Ks in platelet function begins to develop, therapeutic targeting of these enzymes in thrombosis becomes a new consideration. As for Class I PI3Kβ, available evidence suggests selective inhibition of Class II PI3KC2α might offer a new antithrombotic approach, and indeed early data from the Hamilton group at Monash University suggests PI3KC2α inhibitors may prove to be potent anti-thrombotics with a promising safety profile, with their lead compound MIPS-19416 producing an antithrombotic effect that may be largely independent of canonical platelet activation, producing similar observations to studies with PI3KC2α-deficient mice (ISTH Academy. Moon M. July 10, 2019; 274013; OC 78.3). Class II PI3K inhibition has also been proposed as a therapeutic approach in cancer and diabetes [165, 166]. With most of our current understanding of Class II PI3K function in organismal physiology coming from mouse gene targeting, the ongoing development [127–129, 165] of selective Class II PI3K inhibitors as tools will allow an improved understanding of the intricate roles and regulation of these enzymes in humans, and provide a better perspective of whether they may be useful and viable therapeutic targets in human disease. Similarly, given the defect in thrombosis observed with loss of VPS34 in platelets, without an impact on haemostasis, pharmacological inhibition of Class III PI3K has been proposed as a novel anti-thrombotic approach [147, 148], and may also be of benefit for cancer and diabetes [133, 167, 168]. This suggests exciting new clinical opportunities for PI3K inhibition in thrombosis although, as for Class I PI3K inhibitors, whether drugs targeting Class II or Class III PI3Ks could have an acceptable safety and efficacy profile given the importance of these enzymes in normal development and physiology in such a broad range of tissues, and the complexity of PI3K signalling, remains unclear. More generally, major barriers to the development of novel antithrombotic drugs include the challenge of establishing good preclinical models that can adequately mimic the human disease setting, and the relatively high bar of deriving novel therapeutic strategies and candidates that are a sufficient improvement on existing drugs to warrant interest from the pharmaceutical industry, prescribers and patients.

## PI3Ks in other aspects of cardiovascular disease

Beyond platelet function, PI3Ks are implicated in various other aspects of cardiovascular physiology and disease, including atherosclerosis, hypertension, angiogenesis, heart disease and myocardial infarction. Coronary heart disease is commonly associated with atherosclerosis, whereby thrombosis can lead to acute myocardial infarction and sudden cardiac death [169]. PI3Kγ appears to play a role in the pathogenesis of atherosclerosis, and pharmacological inhibition of this Class I PI3K isoform with AS605240 was shown to significantly reduce early atherosclerotic lesions in Apolipoprotein E (ApoE)-null mice, and attenuate advanced atherosclerotic lesions in LDL receptor-deficient mice [170]. PI3Kγ levels are elevated in mouse and human atherosclerotic lesions and the function of this Class I isoform in the haematopoietic lineage supports atherosclerotic progression via roles in macrophage and T cell infiltration, and plaque stabilization [170]. Furthermore, Chang et al. [171] confirmed the role of PI3Kγ in macrophage activation in response to oxidized low-density lipoprotein, inflammatory chemokines and angiotensin II, and also demonstrated that p110γ deficiency leads to a significant reduction in the size of atherosclerotic plaques in ApoE-deficient mice. In addition, GM-CSF-differentiated mouse macrophages become foam cells by PI3Kγ-dependent fluid-phase pinocytosis of LDL [172]. PI3Kγ may therefore represent a therapeutic target in atherosclerosis, as may other PI3K isoforms involved in various cell types implicated in this condition [104], and the relatively restricted expression of PI3Kγ in particular could limit unwanted effects in other tissues [173].

Hypertension is a strong risk factor for cardiovascular disease, and since PI3Ks have roles in the regulation of vascular tone they may represent therapeutic targets in this context. Mice lacking PI3Kγ are protected from angiotensin II-induced hypertension, due to a role for this Class I PI3K in smooth muscle contraction via signalling pathways involving RAC-driven ROS production, and AKT-driven extracellular calcium entry via L-type calcium channels [174]. As such, loss of PI3Kγ function in vivo decreases peripheral vascular resistance via a vasorelaxing effect mediated by loss of pressure-induced AKT phosphorylation and impaired plasma membrane trafficking of the α1C L-type calcium channel in smooth muscle [175]. In support of this, a single nucleotide polymorphism (SNP) in a region flanking the p110γ (PIK3CG) gene locus in humans was shown to influence pulse pressure and mean arterial pressure, and potential risk of cardiovascular events including hypertension, coronary heart disease and stroke risk scores [176]. PI3Kγ’s role in both hypertension and (the often associated) inflammation means inhibitors of this isoform might be attractive therapeutic candidates via both anti-hypertensive and anti-inflammatory properties [173, 176]. Other Class I PI3K isoforms may also hold roles in hypertension, with p110δ expression being upregulated in aortas of hypertensive rats [177], while Class II PI3KC2α appears to regulate vascular smooth muscle contraction and play a role in spontaneous hypertension in rats via a Ca^2+^-PI3KC2α-RHO axis [178–180].

As discussed earlier, both Class I PI3Kα and Class II PI3KC2α support angiogenesis through roles in endothelial cell signalling and function, including transduction of VEGFR signalling and coupling to RHOA to facilitate cell migration [40, 105]. The Class I PI3Kβ and γ isoforms also support endothelial cell migration in response to GPCR agonists such as S1P, and PI3Kγ plays a role in endothelial progenitor cells to support neovascularisation and reperfusion after ischaemia [152, 181, 182]. Modulation of PI3K function may therefore offer therapeutic opportunities to regulate vascular and tissue regeneration after ischaemic damage in cardiovascular disease states. Interestingly, it has been observed that low doses of PI3K inhibitors can improve vascular function [183–185], which might be of value in cardiovascular disease. The studies making these observations were focussed on the impact of PI3K inhibitors on the tumour vasculature in cancer, and suggest that the vascular effects of these drugs can be used to improve drug delivery to tumours while also facilitating immune cell recruitment [183–186]. In contrast, in line with the angiogenesis phenotypes of mice with PI3K deficiency, the potential value of PI3K inhibitors used at higher doses in cancer is via their inhibition or eradication of the tumour vasculature [186]. These effects may be via direct action of the inhibitors against PI3K in endothelial cells, or indirectly via action on other tumour cells [186], myeloid cells [182], and even platelets [187]. Thus, consideration of the intersection between the importance of PI3Ks and their inhibition in cardiovascular (patho)physiology and in cancer may be of value.

In addition to the vasculature, platelets and immune cells, PI3Ks’ various roles in cardiac tissue suggest inhibitors might be of direct value in heart disease. Class I PI3Kα is important for both cardiac development and adult cardiac physiology, controlling cardiomyocyte cell size [188], physiological cardiac growth [189], and thus overall heart size [190]. Furthermore, PI3Kα activity protects the heart against myocardial infarction-induced heart failure [191], can improve the function of a failing heart, is important for exercise-induced cardioprotection [192], and can protect against heart disease in response to dilated cardiomyopathy and acute pressure overload [193, 194]. PI3Kα also negatively regulates gelsolin-activity to suppress gelsolin’s actin-severing activity that contributes to cardiac remodelling in heart failure [195]. PI3Kα’s role in mediating insulin signalling to L-type calcium channels to regulate calcium currents in cardiac myocytes is a key mechanism underpinning the importance of this Class I PI3K isoform in the heart, while both PI3Kα and PI3Kβ support cardiac structure and organisation via regulation of junctophilin-2 localisation and T-tubule organisation [152, 196, 197]. Inhibition of PI3Kα would therefore appear to be detrimental to cardiac development and function and may also have acute detrimental effects such as atrial fibrillation [198]. In contrast, inhibition of PI3Kγ may be cardioprotective and thus a more promising approach for heart failure [152]. Indeed, while PI3Kγ holds a key role in normal cardiac physiology as a scaffolding protein for protein kinase A and phosphodiesterases [199–201], the importance of its catalytic activity is more apparent in the disease setting. p110γ expression is upregulated in congestive heart failure, and its pharmacological inhibition improves contractility in failing hearts by preventing a reduction in β-adrenergic receptor density, with mice expressing a kinase dead form of p110γ showing protection from ventricular modelling and failure caused by pressure overload [152, 199, 201]. In addition to a role for PI3Kγ in regulating β-adrenergic receptors and contractility in cardiomyocytes, this Class I isoform likely also functions in leukocytes to regulate inflammatory signalling in heart failure [152, 202]. In agreement with this, the benefits observed with administration of PI3Kγ inhibitors in animal models of heart disease and failure appear to be dependent on both cardiac contractility and immune cell infiltration [152]. In addition, Class III PI3K’s role in the transition of cardiac hypertrophy to heart failure might suggest inhibition of VPS34 to be of clinical value in this context [203], but demonstration that VPS34 prevents hypertrophic cardiomyopathy by regulating myofibril proteostasis, and that its loss leads to cardiomegaly and decreased contractility, suggests this is unlikely to be a beneficial therapeutic approach [139, 140]. Class II PI3KC2α is also required for cardiac looping during embryonic development [107].

The value of PI3K inhibition for myocardial infarction (MI) remains unclear. The Class I PI3Kδ/γ inhibitor, TG100-115, entered phase I and II clinical trials for acute MI, with observation that it provided cardioprotection in animal models by reducing infarct development and preserving myocardial function even when administered up to 3 h after myocardial infusion [204, 205]. However, selective PI3Kγ inhibition with AS605240 led to an increased infarct size with defective reparative neovascularisation and impaired recovery of left ventricular function in a mouse model of MI, which was supported by the use of PI3Kγ-deficient mice [206]. Furthermore, Haubner et al. [207] reported a protective role for PI3Kγ in myocardial ischaemia–reperfusion injury in mice, mediated via a kinase-independent mechanism. Similarly, PI3Kα has a cardioprotective role in ischaemia–reperfusion injury to limit myocardial infarct size via inhibition of mitochondrial permeability transition pore opening, thus suggesting that promoting, rather than inhibiting, PI3Kα would be preferable in this setting [208]. PI3Kβ also has cell-specific effects in the ischaemic heart, with PI3Kβ activity being protective against myocardial ischaemic injury in cardiac myocytes, while loss of PI3Kβ activity in endothelial cells leads to cardioprotective effects [209].

## The cardiovascular system as an unintended target of PI3K inhibitors

In addition to the consideration of PI3Ks as therapeutic targets for cardiovascular disease, it is clearly also important to consider the significance of their inhibition in cardiovascular tissues as an unintended consequence of PI3K inhibitor administration in other settings, such as oncology. Indeed, at present the predominant focus of PI3K inhibitor development is cancer, with small molecules targeting all Class I PI3K isoforms (either individually or non-selectively) currently in clinical trials, and Class II and Class III PI3K inhibitors being considered for future promise in this setting. From the discussion above, it is clear that PI3K inhibition has the potential to lead to unwanted cardiovascular events, including arrhythmia and cardiac remodelling [198, 210–215]. In some scenarios, the benefits may outweigh the risks, and cardiotoxicity may not apply to all PI3K inhibitors or dosing regimes, but careful cardiovascular monitoring of patients on PI3K-targeted therapy should be in place, particularly for compromised individuals. The authors refer the readers to an excellent discussion by McLean et al. [210] covering this topic, which includes suggestions for strategies to optimize the benefit:risk ratio.

## Conclusions and future perspective

There is now considerable evidence of roles for the PI3K isoforms in various aspects of cardiovascular physiology and disease. This implies these enzymes might prove to be valuable therapeutic targets in contexts such as thrombosis, atherosclerosis and heart failure. In particular, their key roles in platelets suggest Class I, II and III PI3Ks might all be valid anti-thrombotic targets, with the aim of inhibiting thrombosis with limited effect on normal haemostasis. However, it is important to reflect on lessons learned so far in the pursuit of Class I PI3K inhibitors, particularly in the field of cancer, where issues with toxicity, lack of efficacy, and a still limited appreciation of the complexity, redundancy and cell type-specificity of cell signalling has led to much disappointment. It seems clear that in any given cardiovascular context, multiple PI3K isoforms are operating, across multiple cell types, often with both positive and negative roles. Furthermore, given emerging evidence of their important organismal roles, it appears likely that attempts to therapeutically target the Class II and Class III PI3Ks may face similar challenges to those met for Class I PI3K, particularly for the broadly expressed isoforms where managing unwanted inhibition in off-target tissues could be a major challenge.

Nevertheless, the often critical importance of the PI3Ks in various aspects of physiology and disease means they continue to be attractive targets for the development of drugs, and progress continues to be made with innovative new strategies. Indeed, for cancer, while the concept of PI3K inhibition as a monotherapy directly targeting solid tumours has faced challenges [186, 216, 217], value may still come through the use of specific dosing regimes (e.g. higher, but intermittent, dosing) [218], through the combination of PI3K inhibitors with other drugs (e.g. alpelisib/piqray with fulvestrant) [219], through the development of p110α mutant-selective agents (e.g. taselisib) [220], via dietary and pharmacological approaches [221], through understanding the effect of PI3K inhibition on cells beyond the cancer cells themselves (e.g. idelalisib) [186, 217], and through new drug delivery approaches [222]. Time will tell whether these approaches permit PI3K inhibition to be a viable long term therapeutic strategy in cancer, and whether this progress will benefit other conditions such as thrombosis, immune and inflammatory states, and PI3K-related syndromes (e.g. PIK3CA-related overgrowth spectrum (PROS) [223]) with an acceptable safety profile. Platelets and leukocytes may hold more potential for targeting with PI3K inhibitors in comparison to other cell types in more complex, heterogeneous, less accessible, solid tissues or tumours, and the greatest value of PI3K inhibitors may prove to be in acute clinical settings where a therapeutic window may be easier to find. It may be that some inhibitors originally developed for oncology prove to be of more value in other settings. If targeting the PI3K isoforms themselves continues to prove challenging, considering other elements in the PI3K pathway may yet be of value. For example, inhibition of phosphoinositide effectors downstream of the PI3Ks, if druggable, may offer greater functional selectivity and/or target cell selectivity than their upstream masters, to provide drugs with reduced unwanted effects on other PI3K-regulated pathways and tissues. Ultimately, as ever, safer and more efficacious drugs will come from a more detailed understanding of the function and regulation of the individual PI3K isoforms, and their interplay with each other and other cellular effectors across multiple cell types in complex in vivo settings of human health and disease.

## Data Availability

Not applicable.

## Abbreviations

PI3K: phosphoinositide 3-kinase
PtdIns(3,4,5)P_3_: phosphatidylinositol (3,4,5)-trisphosphate
PtdIns(3,4)P_2_: phosphatidylinositol (3,4)-bisphosphate
PtdIns(4,5)P_2_: phosphatidylinositol (4,5)-bisphosphate
PtdIns(3)P: phosphatidylinositol (3)-monophosphate
GEF: guanine nucleotide exchange factor
GAP: GTPase accelerating protein
SH2: Src homology 2 domain
RBD: Ras-binding domain
GPCR: G protein-coupled receptor
